# Elaborating on the assessment of the risk of bias in prognostic studies in pain rehabilitation using QUIPS—aspects of interrater agreement

**DOI:** 10.1186/s41512-019-0050-0

**Published:** 2019-03-07

**Authors:** Wilhelmus Johannes Andreas Grooten, Elena Tseli, Björn Olov Äng, Katja Boersma, Britt-Marie Stålnacke, Björn Gerdle, Paul Enthoven

**Affiliations:** 1 Department of Neurobiology, Care Sciences and Society, Division of Physiotherapy, Karolinska Institutet, Huddinge, SE-141 83 Sweden; 2 Allied Health Professionals Function, Functional area Occupational Therapy and Physiotherapy, Karolinska University Hospital, Stockholm, SE-171 76 Sweden; 3 School of Education, Health and Social Studies, Dalarna University, Falun, SE-791 88 Sweden; 4 Center for Clinical Research Dalarna - Uppsala University, Falun, SE-791 82 Sweden; 5 School of Law, Psychology and Social Work, Örebro University, Örebro, SE-701 82 Sweden; 6 Department of Community Medicine and Rehabilitation, Rehabilitation Medicine, Umeå University, Umeå, SE-901 87 Sweden; 7 Department of Clinical Sciences, Danderyd Hospital, Karolinska Institutet; Department of Rehabilitation Medicine, Danderyd Hospital, SE-182 88 Stockholm, Sweden; 8 Pain and Rehabilitation Centre, and Department of Medical and Health Sciences, Linköping University, SE-581 85 Linköping, Sweden; 9 Division of Physiotherapy, Department of Medical and Health Sciences, Linköping University, SE-581 85 Linköping, Sweden

**Keywords:** Chronic pain, Inter-rater agreement, Meta-analysis, Prognosis, Rehabilitation, Review, Risk of bias

## Abstract

**Background:**

Many studies have been performed to identify important prognostic factors for outcomes after rehabilitation of patients with chronic pain, and there is a need to synthesize them through systematic review. In this process, it is important to assess the study quality and risk of bias. The “Quality In Prognosis Studies” (QUIPS) tool has been developed for this purpose and consists of several prompting items categorized into six domains, and each domain is judged on a three-grade scale (low, moderate or high risk of bias). The aim of the present study was to determine the interrater agreement of the risk of bias assessment in prognostic studies of patients with chronic pain using QUIPS and to elaborate on the use of this instrument.

**Methods:**

We performed a systematic review and a meta-analysis of prognostic factors for long-term outcomes after multidisciplinary rehabilitation in patients with chronic pain. Two researchers rated the risk of bias in 43 published papers in two rounds (15 and 28 papers, respectively). The interrater agreement and Cohen’s quadratic weighted kappa coefficient (*κ*) and 95% confidence interval (95%CI) were calculated in all domains and separately for the first and second rounds.

**Results:**

The raters agreed in 61% of the domains (157 out of 258), with similar interrater agreement in the first (59%, 53/90) and second rounds (62%, 104/168). The overall weighted kappa coefficient (kappa for all domains and all papers) was weak: *κ* = 0.475 (95%CI = 0.358–0.601). A “minimal agreement” between the raters was found in the first round, *κ* = 0.323 (95%CI = 0.129–0.517), but increased to “weak agreement” in the second round, *κ* = 0.536 (95%CI = 0.390–0.682).

**Conclusion:**

Despite a relatively low interrater agreement, QUIPS proved to be a useful tool in assessing the risk of bias when performing a meta-analysis of prognostic studies in pain rehabilitation, since it demands of raters to discuss and investigate important aspects of study quality. Some items were particularly hard to differentiate in-between, and a learning phase was required to increase the interrater agreement. This paper highlights several aspects of the tool that should be kept in mind when rating the risk of bias in prognostic studies, and provides some suggestions on common pitfalls to avoid during this process.

**Trial registration:**

PROSPERO CRD42016025339; registered 05 February 2016.

**Electronic supplementary material:**

The online version of this article (10.1186/s41512-019-0050-0) contains supplementary material, which is available to authorized users.

## Background

A prognostic factor is any measure associated with a subsequent clinical outcome among people with a given health condition [1, 2]. For example, in patients with chronic pain, those with higher levels of emotional distress have a worse prognosis, e.g. worse functional outcome after rehabilitation [3], while pain intensity in combination with anxiety or depressive symptoms has been identified as important prognostic factors for poor recovery [4]. Prognostic factors may thus distinguish groups of people with a different average prognosis, and by identifying these, prognostic models can be developed to provide important information for health research and guidance for more effective and efficient care [5]. The PROGRESS group (PROGnosis RESearch Strategy) has put forward prognostic studies as an important base of knowledge for clinical decision making, healthcare policy, and discovering and evaluating new approaches to patient management [6]. In studies on rehabilitation of chronic pain, several prognostic factors from different domains (demographic, illness-related, rehabilitation-related, social) have been suggested influencing a large number of outcomes (pain, function, work, quality of life) at the same time. Each year, hundreds of studies in the field of pain rehabilitation investigate different prognostic factors. Frequently, the findings are inconsistent; some authors suggest that a particular factor is prognostic while others suggest the contrary [2, 7, 8]. For this reason, it is important to perform a systematic review (SR) of existing data in order to evaluate the prognostic value of this particular factor. The aim of such SR is thus firstly to look at whether a potential prognostic factor is associated with an outcome and secondly to estimate the strength of the association through a meta-analysis (MA), where all study findings are synthesized and summarized, for example by calculating an average effect size. In this process, it is important to assess the quality of the included papers in order to decide if and to what extent a paper may contribute to the review (and synthesis). This process is called risk of bias assessment (RoB) [9]. If the included papers have methodological flaws, it is difficult to draw any conclusions regarding the existence and strength of any associations and therefore has the PROGRESS group put forward several recommendations to increase the study quality of prognostic studies, the transparency, better reporting, and several other aspects that can be improved, such as improved standardization [2, 6]. RoB assessment, however, is a process of subjective judgment, and since different raters could rate differently, the use of a standardized tool to guide such critical appraisal in conducting systematic reviews is suggested. By using this type of tool, the raters are better equipped to systematically discuss all important aspects of study quality in a more transparent way, which also makes it easier to reach a consensus between all raters.

Cochrane Prognosis Methods Group recommends the use of the Quality In Prognosis Studies (QUIPS) tool to assess RoB in prognostic factor studies [10]. The QUIPS tool uses six important domains that should be critically appraised when evaluating validity and bias in studies of prognostic factors: (1) study participation, (2) study attrition, (3) prognostic factor measurement, (4) outcome measurement, (5) study confounding, and (6) statistical analysis and reporting [11]. The different domains contain between three and seven prompting items to be rated on a four-grade scale (yes, partial, no, unsure). Some of these items are informal and can be answered with a “yes” or “no”, mainly referring to whether essential study information has been reported (e.g. “reasons for loss to follow-up are provided”), but for other items, the rater needs to make a more subjective decision (e.g. “there is adequate participation in the study by eligible individuals”). In a final stage, the rater makes an overall, conclusive judgment of the risk of bias within each domain, based on their ratings of the included items. This risk is expressed on a three-grade scale (high, moderate or low RoB) [11]. Hence, the QUIPS assessment results in six ratings of RoB, one for each domain, which are then compared for interrater agreement.

In the original study by Hayden et al. [11], kappa values evaluating QUIPS were reported to vary from 0.56 to 0.82 (median, 0.75) showing that the raters involved had relatively good interrater agreement. Still, Hayden et al. stated that further reliability testing should be done on a larger, more representative set of studies and tool users, including assessing reliability of individual domain ratings, as well as consensus ratings between groups [11]. To our knowledge, QUIPS has not yet been widely used for RoB assessment of prognostic studies in the field of pain rehabilitation and there are no specific interrater reliability studies on QUIPS performed. Hence, the aim of the present study was to determine the interrater agreement of the risk of bias (RoB) assessment in prognostic studies using QUIPS and to elaborate on our experiences.

## Methods

This paper is divided into one part on interrater agreement and one part elaborating on the experiences of using QUIPS. We performed a meta-analysis of early (baseline) prognostic factors in patients with chronic pain, which could influence multiple long-term outcomes after multidisciplinary rehabilitation (MDR) [12]. The aim of the meta-analysis was to find out what factors were associated with defined outcomes (and the level of evidence), rather than exploring a hypothesis of one specific prognostic factor for one specific outcome. Hence, papers published between 1994 and 2014 with various longitudinal study designs were included: prognostic studies, register data studies, randomized controlled intervention studies, non-controlled studies, etc. The papers were predominantly observational, non-controlled intervention studies that included patient information (prognostic factors) of the population of interest at baseline, together with outcome data (pain, function, work status, quality of life, etc.) at 6 months or more after baseline and subsequent MDR (see Additional file 1). In the research team, three authors with expertise within MDR worked on the final selection of potential papers. Thereafter, two researchers with expertise within epidemiology and prognostic studies assessed the RoB of the included papers independently following a randomization scheme. A third junior researcher was also involved in this process, but in the present paper, the agreement between the two senior researchers is the focus of interest.

### QUIPS rating procedure

To assess the RoB of the included papers, the QUIPS electronic spreadsheet (in MS Excel) as provided by Hayden et al. [11] was used. The two raters independently inserted relevant information from each paper in their own electronic assessment spreadsheet. The raters were able to make specific notes on quality issues for each prompting item. Each rater used only one MS Excel sheet for each paper, although several prognostic factors and outcomes could have been evaluated in the same paper. Hence, for each domain, the raters judged the six domains on the *overall* quality of the paper. The authors’ notes were used later during our peer-group discussions for reaching agreement (on the overall quality of the study) when summarizing the level of evidence for every separate prognostic factor.

For QUIPS, there are no rules available that indicate how the researcher should classify the overall RoB of a paper or, in other words, how to summarize the RoB of all six domains into one overall rating on paper level, but it is recommended against computing summated scores for overall study quality [14]. In systematic reviews and/or meta-synthesis, however, it is recommended to include a table of included papers in which each paper is classified as having high, moderate or low RoB. It became thus evident that some sort of categorization of the papers was necessary to describe the included papers for our synthesis after finalizing all RoB assessments [3]. We based this categorization on the following criteria: If all domains were classified as having low RoB, or up to one moderate RoB, then this paper was classified as low RoB (green). If one or more domains were classified as having high RoB, or ≥ 3 moderate RoB, then this paper was classified as high RoB (red). All papers in between were classified as having moderate RoB (yellow). This categorization was a result of a continuous discussion between the authors.

### Procedure

An initial pilot screening of five non-included papers was used to prepare the raters involved for the use of QUIPS, reaching a consensus on how to judge the different prompting items and how to summarize them in order to judge a domain. The two experienced senior researchers independently rated the RoB in 43 of the included papers in two separate rounds with a mid-point discussion in-between. The discussions were used to resolve any discrepancies and to achieve 100% consensus for the papers considered in the first and second rounds, including 15 and 28 papers, respectively. Moreover, any detected vagueness in the “key-list” were further resolved during this mid-point discussion.

### Statistical analyses

#### Interrater agreement

All RoB domains in all papers (*n* = 43) were separately judged by the raters as having low, moderate or high RoB. Agreement was defined as the two experienced senior researchers scoring alike on a domain, while maximal disagreement was defined as one rater scoring low RoB and the other scoring high RoB. All other cases of disagreement were defined as partial disagreement.

To assess whether the two experienced senior researchers scored the QUIPS domains equally, the interrater agreement was investigated to determine the consistency between the raters. The rate of agreement was calculated as a percentage by the number of domains judged alike by the two raters divided by the total number of domains (*n* = 258; i.e. 43 papers × 6 domains) before reaching consensus. This was performed across all papers, but also for the first and second rounds separately (*n* = 90 and *n* = 168, respectively). An online kappa calculator was used to calculate Cohen’s kappa with quadratic weighting (http://vassarstats.net/kappa.html) for all domains combined and for each separate domain across all 43 papers, but also for the two rounds separately. The coefficient was interpreted as follows: *κ* = 0.00–0.20, no agreement; *κ* = 0.21–0.39, weak agreement; *κ* = 0.40–0.59, minimal agreement; *κ* = 0.60–0.79, moderate agreement; *κ* = 0.80–0.90, strong agreement; and *κ* > 0.90, almost perfect agreement [13].

An online test of proportion calculator (https://www.medcalc.org/calc/comparison_of_proportions.php) was used to test if there were any differences in overall agreement between the raters, between the different rounds and between the papers with high and low RoB.

## Results

### Interrater agreement

In four out of the 43 papers (9.3%), there was full initial agreement between the two experienced senior researchers in all six domains. The raters judged similarly in five domains in 10 papers (23.2%); they disagreed four times in domain 6, three times in domain 1, and one time in domains 2, 3 and 5. The raters judged similarly in four domains in nine papers (20.9%); they disagreed five times in domain 5, four times in domain 3, three times in domains 1 and 3, and two times in domain 4. In 12 papers (27.9%), three domains were rated similarly; most disagreements occurred in domain 6 (nine times), followed by seven times in domain 3, six times in domain 5, five times in domain 1 and 2, and four times in domain 4. In six papers (14.0%), two domains were rated similarly; most agreement was found for domain 5 (four times) and domain 3 (three times), while the raters agreed two times in domain 6 and one time in the remaining domains each. In the remaining two papers, only one domain was judged similar (4.7%). For high-quality papers, those classified with low RoB, the agreement percentage was somewhat higher (88/133 domains = 66.2%) than for papers with high RoB (22/38 domains = 57.9%); *p* = 0.3475.

All in all, the results show that in 23 out of 43 (53.5%) of the papers, at least four domains were scored similar by the two raters (see Additional file 2).

Table 1 shows the two raters’ initial judgments for all 258 domains (43 papers × 6 domains). The raters agreed in 157 domains (60.9%). In the first round, the raters judged alike in 58.9% (53/90) of the domains, and in the second round, in 61.9% (104/168). Although no differences between the rounds concerning % agreement were seen, both raters had a higher proportion of prompting items classified as high RoB in the second round compared to the first round.

**Table 1:** Comparison of risk of bias judgment for all papers and for rounds 1 and 2 separately

All (43 papers)		Rater 2			Total
		Low	Moderate	High	
Rater 1	Low	88 (34%)	39 (15%)	6 (2%)	133 (52%)
	Moderate	36 (14%)	47 (18%)	4 (2%)	87 (34%)
	High	6 (2%)	10 (4%)	22 (9%)	38 (15%)
Total		130 (50%)	96 (37%)	32 (12%)	258 (100%)
Round 1 (15 papers)					
Rater 1	Low	32 (36%)	10 (11%)	3 (3%)	45 (50%)
	Moderate	17 (19%)	18 (20%)	1 (1%)	36 (40%)
	High	2 (2%)	4 (4%)	3 (3%)	9 (10%)
Total		51 (57%)	32 (36%)	7 (8%)	90 (100%)
Round 2 (28 papers)					
Rater 1	Low	56 (33%)	29 (17%)	3 (2%)	88 (52%)
	Moderate	19 (11%)	29 (17%)	3 (2%)	51 (30%)
	High	4 (2%)	6 (4%)	19 (11%)	29 (17%)
Total		79 (47%)	64 (38%)	25 (15%)	168 (100%)

In 4.6% of the domains (12/258), the raters disagreed maximally. Maximal disagreement was not caused by one of the raters judging consistently lower or higher RoB than the other; hence, there was no systematic difference in rating style. The overall quadratic weighted kappa coefficient (kappa for all domains and all papers) was interpreted as a “weak agreement”, *κ* = 0.475 (95% CI = 0.358–0.601). “Minimal agreement” was found for the first round, *κ* = 0.323 (95% CI = 0.129–0.517), and the kappa improved somewhat in the second round to weak agreement, *κ* = 0.536 (95% CI = 0.390–0.682).

The Kappa calculations for the separate domains showed that minimal agreement was found for domains 1, 3, 4 and 6; weak agreement was found for domain 5 “study confounding”; and “moderate agreement” was found for domain 2 “study attrition” (Table 2).

**Table 2:** Interrater agreement for all papers and for the two rounds separately

	All papers (43 papers)		Round 1 (15 papers)		Round 2 (28 papers)	
	*κ* ^1^	95% CI^2^	*κ* ^1^	95% CI^2^	*κ* ^1^	95% CI^2^
1. Study participation	0.356	0.063–0.669	− 0.023	− 0.041–0.594	0.475	0.100–0.851
2. Study attrition	0.647	0.353–0.771	0.400	0.000–0.835	0.714	0.426–1.000
3. Prognostic factor measurement	0.384	0.044–0.723	0.852	0.544–1.00	0.028	− 0.453–0.612
4. Outcome measurement	0.358	0.072–0.645	0.211	0.00–0.660	0.443	0–095-0.792
5. Study confounding	0.526	0.269–0.784	0.561	0.00–1.00	0.481	0.186–0.776
6. Statistical analysis and reporting	0.364	0.077–0.651	− 0.070	0.321–0.486	0.533	0.176–0.627
Overall	0.475	0.358–0.601	0.323	0.129–0.517	0.536	0.390–0.682

^1^Quadratic weighted kappa (*κ*)

^2^95% Confidence Interval (95% CI)

### Elaboration on QUIPS

With the intention to improve future inter-rater agreement on RoB assessment, we elaborate below on the prompting items in the QUIPS tool that should be discussed before performing RoB assessment in a systematic review. This is in line with Hayden et al., who encouraged clear specification of the tool items, as this will probably improve interrater agreement [11]. Table 3 gives some of the specific questions that raters could use in their discussion on the prompting items before and during the review process. Using these questions, raters could systematically and objectively reach a consensus.

**Table 3:** Suggestions for questions and comments on QUIPS risk of bias domains and our team’s proposed answers

RoB domain and corresponding “prompting items”	Suggestions of questions and comments where consensus is needed.	Our team’s answers to the questions from a pain rehabilitation perspective, which will produce a “yes”
1. Study participation		For a “yes”, …
a) Adequate participation in the study by eligible persons.	a) What is adequate?	a) … the participation should be at least 67%. Our experience is that in this field of those being assessed for participation not all are eligible and therefore we permitted a somewhat lower participation rate compared to what can be used in other fields [12].
b) Description of the source population or population of interest	b) When is the lack of such a description related to bias?	b) This prompting item was not taken into account, since only a few studies reported information of the source population.
c) Description of the baseline study sample	c) What is important to know about the study sample?	c) … basic information available regarding gender, age, socioeconomic status together with some disease-related information (pain, disability, comorbidities) and information on relevant outcome data .
d) Adequate description of the sampling frame and recruitment	d) What is the minimal information on the sampling frame and recruitment procedure and what is an “incorrect sampling frame”?	d) … information available on the patients’ recruitment (from which health service) together with a description of how the data collection was performed.
e) Adequate description of the period and place of recruitment	e) What is the minimal information needed?	e) … there should be information available on the beginning and end of the data collection, the setting, and the name of a geographical place or hospital. Additionally, it could be more important to know if the patient was filling in questionnaires with/without influence of the caregivers.
f) Adequate description of inclusion and exclusion criteria	f) What is the minimal information needed?	f) … at least 1 inclusion and 1 exclusion criterion should be given.
2. Study attrition		For a “yes”, …
a) Adequate response rate for study participants	a) What is adequate?	a) … the response rate should be at least 67%. The experience from this field is that due to the relative long follow-up time (6mo) and differences in follow- up time (12, 18mo), a relative larger loss of participants at follow-up compared to other fields could be expected.
b) Description of attempts to collect information on participants who dropped out	b) What is the minimal information needed?	b) … information available on the methods and timing.
c) Reasons for loss to follow-up are provided	c) What is the minimum information needed?	c) … any information available on the reasons for drop-outs
d) Adequate description of participants lost to follow-up	d) What is adequate? Do we need to see the analyses or is it enough with one sentence?	d) … any information available on gender, age and disease-related information for drop-outs.
e) There are no important differences between participants who completed the study and those who did not	e) What is important? Demographic differences (hampering generalization) or illness-related differences (hampering validity?)	e) … there should be no differences between the participants and non-participants in regard to demographic and illness-related variables, such as levels of pain intensity, disability, absence from work.
3. Prognostic factor (PF) measurement		For a “yes”, …
a) A clear definition or description of the PF is provided	a) What is the minimal information needed?	a) … there should be a clear definition of the PF, e.g. information on which question(s) were used, how the data was collected, how the variable was constructed, etc.
b) Method of PF measurement is adequately valid and reliable	b) What is adequate? When is an instrument valid/reliable?	b) … there should be a reference to a reliability/validity study or information on these features in the paper and this paper should cover the field of chronic pain rehabilitation. In the field of rehabilitation of chronic pain we suggest that when different prognostic factors are included with different RoB, this issue should be noted and solved in the synthesis phase of the SR/MA (e.g. making decisions of excluding those invalid instruments, or downgrading the level of evidence).
c) Continuous variables are reported or appropriate cut points are used	c) What is appropriate?	c) … the cut-offs used should NOT be based on distribution of the data, but on established cut-offs in the field of chronic pain rehabilitation.
d) The method and setting of measurement of PF is the same for all study participants	d) Is it OK if not the same, but two reliable measurements are used?	d) …the PF should be the same, but also could be different for different study participants if both measures are reliable (e.g. VAS or NRS when measuring pain). However, both instruments should be valid for the use in the field of chronic pain.
e) Adequate proportion of the study sample has complete data for the PF	e) What is adequate?	e) … there should be at least 67% available with complete data. It is important also to check if there is different data available for different prognostic factors measured simultaneously, which could indicate differential loss-to follow-up.
f) Appropriate methods of imputation are used for missing PF data	f) What is appropriate?	f) … there should be some kind of imputation, but even if no imputation was done, it could be a “yes” if at least 67% of the study sample had complete data.
4. Outcome measurement		For a “yes”, …
a) A clear definition of the outcome is provided	a) What is the least information needed?	a) … there should be a clear definition of the outcome measure available, e.g. information on the question(s) used, how the data was collected, how the variable was constructed, etc. In the field of rehabilitation of chronic pain we suggest that the IMMPACT recommendations on outcome measures should be used [13].
b) Method of outcome measurement is adequately valid and reliable	b) What is adequate? When is an instrument valid/reliable?	b) … there should be a reference to a reliability/validity study or information on these features in the paper. Note the population on which the reliability/validity study was performed should correspond to the population of interest, in this case patients with chronic pain.
c) The method and setting of outcome measurement is the same for all study participants	c) Is it OK if not the same, but two reliable measurements are used?	c) … the outcome measures should be the same, but could also be different for different study participants if both measures are reliable (e.g. VAS or NRS when measuring pain). However, both instruments should be valid for the use in the field of chronic pain.
5. Study confounding		For a “yes”, …
a) All important confounders are measured	a) Which confounders are of importance?	a) … there should be at least one confounder taken into account. However, in a broad review in the field of chronic pain with multifactor associations between prognostic factors and outcome it is difficult to know if one should use the results of the multivariate analyses in the MA or if the univariate results should be used. On one hand, the univariate analyses could be confounded by other factors, hence a multivariate analysis should be the appropriate choice. However, in a multivariate analysis, the PF of interest could have been removed or the results could have been altered through interaction of other factors, leading to a lack of data or overestimation of the PF of interest in a MA. In our current study on prognostic factors for functioning in patients with chronic pain we included both uni- and multivariate estimates in the MA, but indicated this in the forest plots and used sensitivity analyses for re-analysing the results.
b) Clear definitions of the important confounders measured are provided	b) What is the minimal information needed?	b) … there should be a clear definition of the confounder measure available, e.g. information on the question(s) used, how the data was collected, how the variable was constructed, etc.
c) Measurement of all important confounders is adequately valid and reliable	c) What is adequate? When is an instrument valid/reliable?	c) … there could be a reference to a reliability/validity study or information on these features in the paper and the confounder should be valid for the use in the field of chronic pain.
d) The method and setting of confounding measurement are the same for all study participants	d) Is it OK if not the same, but two reliable measurements are used?	d) … the confounder measures should be the same, but could also be different for different study participants if both measures are reliable (e.g. VAS or NRS when measuring pain).
e) Appropriate methods are used if imputation is used for missing confounder data	e) What do we accept as an appropriate method when handling missing data?	e) … there could be any kind of imputation, but even if no imputation was done, it could be a “yes” if at least 67% of the study sample had complete data.
f) Important potential confounders are accounted for in the study design	f) What do we accept as an appropriate study design?	f) … there should have been some kind of randomization or confounders included in the analyses. Since blinding of researchers and patients to treatment is nearly impossible in the field of chronic pain rehabilitation, we accepted randomization only.
g) Important potential confounders are accounted for in the analysis	g) If not, do we judge here or on point 6c) or on both points?	g) see point f) and point 6c)
6. Statistical Analysis and Reporting		For a “yes”, …
a) Sufficient presentation of data to assess the adequacy of the analytic strategy	a) What is sufficient?	a) … there should be enough information available to understand the statistical methods applied, so that the rater can determine whether the methods used were correct.
b) Strategy for model building is appropriate and is based on a conceptual framework or model	b) What is appropriate? If there is no model described, is there a risk of bias?	b) We decided that this point was NOT to be considered, but more emphasis was put on point 6a)
c) The selected statistical model is adequate for the design of the study	c) What is adequate?	c) … there should be some form of statistical analyses description available, resulting in information on the effect of the PF on the outcome.
d) There is no selective reporting of results	d) Is there a need to check pre-publications such as protocols for this information?	d) … all variables (outcomes and PF) that are described in the method section should be included in the result section with words or in numbers (tables, figures).

*MA* meta-analysis, *VAS* visual analogue scale, *NRS* numeric rating scale

For all domains, it is important to decide the weight of each item, e.g. concerning the prompting item on the source population in the domain “study participation”. Only a few papers in our sample reported any data in this regard, so we decided not to put much emphasis on this prompting item, since it is unclear how the OR of a prognostic factor can be different between participants and eligible non-participants if the source population is not presented.

On the prompting items relating to “participation and attrition”, we found it important to use cut-offs in order to ensure consistency. In our analyses, we used an arbitrary cut-off point of at least 67% for both participation and attrition rates as acceptable. This factor probably increased the consistency in the second round. In order to reach consistency between the raters for many of the prompting items, it is crucial to reach a consensus on what is meant by “adequate” and how much information is needed to produce a yes.

Within our research team, we discussed how to judge the RoB on the prompting item “The method and setting of outcome measurement is the same for all study participants” when two different but both adequate measures were used. For instance, is there an increased risk of bias if patients’ pain intensity levels were measured both using a visual analogue scale (VAS) and a numeric rating scale (NRS)? Both measures are considered valid and reliable and measure the same construct “pain intensity”. It is therefore difficult to know if this introduced a systematic error in the OR estimates.

Regarding the domains “confounding” and “statistical analyses”, it is important that the raters are familiar with epidemiological methodological issues and statistical analyses, which was the case in the current study. Despite this, the raters sometimes differed in their RoB judgments. One explanation is that different experts prefer different statistical methods, and there is no golden standard for which method is the most optimal method that should be used. It is therefore important to have a discussion on this topic to reach consensus on which study design, regression models, and imputation methods are acceptable and which are not.

Sometimes, we found it difficult to know “where” (in which domain) to rate a methodological flaw and/or if this flaw should be counted one or two times. For example, when a paper provided an odds ratio (OR) for a prognostic factor without reporting if the OR was adjusted for confounding, one rater could rate a “high RoB” in the domain of “statistical analysis and reporting” (poor reporting), while the other could rate high RoB in the domain of study confounding (presuming that the data was not adjusted for confounders since it was not mentioned). Moreover, we used neither any specific guidelines nor any prior criteria about how many “nos” on specific prompting items resulted in to moderate or high RoB for that domain, but the raters felt that the judgment was not that hard. If for example the item “recruitment period” was classified as no but the other five prompting items in the “participating domain” were classified as a yes, then the overall rating on this item was “low RoB”, while if the prompting item on participating rate was classified as a no, the RoB would be classified as a high RoB in this domain.

As a final comment on the prompting item in the domain of “Statistical analyses and reporting - There is no selective reporting of results”, it is important to reach consensus on whether or not to check published protocols or other pre-publications for selective reporting of the outcomes and prognostic studies, or if the paper should be judged without this additional knowledge.

## Discussion

The results of our study showed that the raters agreed in 61% of the domains, with similar interrater agreement in the first and second round. The overall weighted kappa coefficient was weak, and suggestions for improvement were provided.

Hayden et al. [11] reported kappa values varying from 0.56 to 0.82 (median 0.75) from nine review teams on 205 studies. Compared to these studies, our kappa values were somewhat lower, which might be due to various causes. A limitation when comparing the present study with the study of Hayden et al. [11] is that all studies quoted in Hayden et al. used a previous version of the tool instead of the currently recommended QUIPS tool applied in our study. Some studies [14, 15] used a previous version of QUIPS, or a previous version combined with items from other tools [16–18]. It is therefore difficult to know to what extent kappa values are influenced by the different versions of the QUIPS tool. Furthermore, in several studies, kappa values were not based on RoB assessment of the six QUIPS domains, but on the prompting items [14, 17–22], which is not recommended [11] since calculating kappa from a large number of items (using a yes/no judgment) compared to a small number of domains (using a three-grade scale) may result in an overestimation of the kappa value. Some studies [15, 21] calculated a total quality score for each study based on all domains, which may not be particularly informative, as it ascribes equal weight to each of the nominated items, and the method is therefore not recommended [11, 23]. There are also large differences between the RoB of different domains, which also suggests that an overall rating based on all domains should not be used. Some reviews included studies with other designs than prognostic prospective longitudinal studies, which also could have influenced the ratings [19, 20].

A number of recent reviews have investigated RoB using the same QUIPS version as the current study [24, 25] and describe the process of involving a third rater in order to reach total agreement. In the present study, this was not necessary because the two raters were able to come to 100% consensus through discussion. Bruls et al. [24] showed, similar to our results, a large variation in kappa values for the different domains (*κ* = 0.13 for domain study confounding and *κ* = 0.82 for domain study participation). den Bakker et al. [25] also found low interrater agreement for the domain study confounding, and these results are somewhat contradictory to our results, which showed the second highest kappa coefficient for this domain (all studies). One reason for this discrepancy could be that the majority of the papers included in our analyses were methodologically flawed in this particular domain. Thirteen papers were rated as high RoB in this domain (compared to only 3 papers in the domain on “prognostic factor measurement”). It should be noted that disagreement between the raters is related to (1) the interpretation of the text (the paper), and (2) the interpretation of the specific prompting items in the domain. It is impossible to separate these two factors, which could make it difficult to speculate on the reasons for discrepancies between various studies.

Several authors [24–26] describe that difficulties to reach agreement are concentrated to some of the QUIPS domains, and the domains that are problematic may depend on the area of research. If the area is complex, it is more difficult to reach an agreement, possibly because the items can be interpreted in different ways. It might be more difficult, as in the current study, to perform RoB assessment on prognostic studies in the field of pain rehabilitation and on patients with chronic pain, due to the well-known diversity of the symptoms, variation in outcomes and other methodological difficulties. Most of the papers included in the present study were classified as having multiple domains with high risk of bias. Additionally, as our review included papers in which several prognostic factors and outcomes were assessed at the same time, it was a methodological challenge to assess the RoB assessment at study level, i.e. to give an overall score. For example, the RoB may vary within a study for different prognostic factors and outcomes, as well as the judgment for assessment of confounding. Using only *one* QUIPS electronic Excel spreadsheet for each paper may have introduced difficulties to rate the level of evidence for each single prognostic factor, which was our overall purpose with the SR/MA. It is therefore important, especially in broad reviews, that researchers à priori decide if the RoB applies at study level or at single factor level.

In our study, we found that the interrater agreement was higher in papers of good quality. Some, but not all, of the reviews reporting strong inter-rater agreement also found that most of the included studies had good methodological quality and low RoB [14, 17]. This is consistent with the findings of Wright et al. [22] and Paxton et al. [27] in which a higher kappa value was found in studies of high quality. Paxton et al. [27], using the same version of QUIPS as in the current study, reported moderate agreement (kappa = 0.7 (95% CI 0.5-0.8), which is somewhat higher than the overall kappa for the present study, but appears to be in line with our results for the second round. One reason for a higher kappa in their study might be that about half of the studies included were classified with low RoB [27], which was a higher percentage than in the current study. It seems that recently published papers have a higher methodological quality than earlier papers, i.e. have lower RoB [25, 27], and our study included many papers that were published more than 10 years ago. One reason for the quality improvement of more recent papers might be the introduction of specific checklists for reporting studies, which may have had a positive effect on the interrater agreement of RoB assessment. Our experience was that assessing the RoB was easier in high-quality papers compared to poorly written papers. In relation to this, differences in study quality between our study and the aforementioned studies could lead to different distributions in the 3 × 3 tables, and since the kappa coefficient is dependent on the prevalence index, the differences in results could also be due to differences in prevalence distribution [28].

We believe that it is important to have regular discussions in the research group on how to interpret the instructions to the prompting items in the QUIPS tool. Since the raters were experienced and senior in the field, both identified the methodological flaws in the studies, but disagreed on where (at what prompting item and domain) to rate the specific methodological drawback (see below). Discussions were needed to rate the importance of the prompting items and also to agree on how many nos on the prompting items in the different domains were needed to rate that domain as “moderate or high risk of bias”. Hayden et al. emphasize the need of modification/clarification of the prompting items for each research question [11]. We agree that it is very important for raters to have several meetings before and during the process. While this flexibility makes it easier to apply QUIPS in different areas, it may also make it more difficult to compare the results of different review studies, even within the same area. Interestingly, the kappa for the domain “prognostic factor” was much lower in the second round compared to the first round, but we could not find any plausible explanation as to why this occurred.

### Methodological considerations

Among the strengths of our study, this paper is unique in several aspects: not only we analysed levels of interrater reliability and elaborated on several aspects of the QUIPS tool, but we also reported on our experiences and the learning process of using this tool in the field of rehabilitation of chronic pain. On the other hand, the number of raters and papers included in our analyses was small, resulting in low prevalence in some of the cells (*n* = 1–3), when performing sub-group analyses. Moreover, the results of this paper could have limited influence on the use of the QUIPS tool when assessing the RoB in prospective studies in other areas, but we believe that our paper can serve to inspire researchers from other fields to work in a more structured manner and avoid the common pit-falls we have highlighted in this paper. As a final comment, it is important to notice that kappa statistics we used showed the level of agreement between the raters, which is not the same as the agreement to the “true RoB”. Due to the subjectivity of the ratings and the lack of a golden standard, there could have been an overestimation of the estimates if both raters make the same error.

### Implications

This study could have some implications for further research. Firstly, we underline the importance of having at least two raters carry out RoB assessment of every paper included in the review, which is not the case in a number of recently published review studies. Secondly, it is important that the raters have initial and continuing discussions throughout the process on how to interpret the instructions, how to define the qualifiers used in the instructions and cut-off points when applicable (e.g. the 67% attrition/participation rate), set for defining “minimal level of adequate”, and, finally, that they agree on how to define differences between moderate and high RoB as well as how to classify the overall RoB. The researchers should keep in mind that the suggestions presented in this study should not be seen as decision trees, but as aspects to discuss when assessing the RoB. Since QUIPS is the main recommended tool for analysing RoB in prognostic studies, it could be of interest to continue refining the tool for example through world-wide workshops including researchers from all fields. Perhaps in the future, as the design and conduct of prognostic studies may differ between fields, some field-specific prompting items could be added as well as some additional guidance for QUIPS. As a first step, it could be of interest to study if the use of the proposed questions influences the overall agreement between raters.

## Conclusions

Despite relatively low interrater agreement, QUIPS proved to be a useful tool for assessing the risk of bias when performing a meta-analysis of prognostic studies in pain rehabilitation, since it demands of raters to discuss and investigate important aspects of study quality. Some items were particularly hard to differentiate, and a learning phase was required to increase the interrater agreement. This paper highlights several aspects of the tool that should be kept in mind when rating the risk of bias in prognostic studies and provides some suggestions on common pitfalls to avoid during this process.

## Additional files

Additional file 1: List of included papers.doc. This Word-file contains references of all 43 studies included in the analyses. (DOCX 16 kb)

Additional file 2: Datasheet.xls. This Excel datasheet includes the ratings of the two included raters on each RoB domain in all 43 studies. (XLSX 18 kb)

## Abbreviations

95%CI: 95% confidence Interval
MA: Meta-analysis
MDR: Multidisciplinary rehabilitation
NRS: Numeric rating scale
OR: Odds ratio
QUIPS: Quality In Prognosis Studies
RoB: Risk of bias
SR: Systematic review
VAS: Visual analogue scale
