# Supplementary material for: Elaborating on the assessment of the risk of bias in prognostic studies in pain rehabilitation using QUIPS—aspects of interrater agreement
Source: Diagn Progn Res. 2019 Mar 7;3:5. doi: 10.1186/s41512-019-0050-0 (PMC6460536; doi:10.1186/s41512-019-0050-0)
Supplement: Supplementary file 1 — List of included papers.doc. This Word-file contains references of all 43 studies included in the analyses. (DOCX 16 kb) [file 41512_2019_50_MOESM1_ESM.docx]

**Records included in the Risk of Bias assessment.**

Bailey, B. E., Freedenfeld, R. N., Sanford Kiser, R., & Gatchel, R. J. (2003). Lifetime physical and sexual abuse in chronic pain patients: Psychological correlates and treatment outcomes. *Disability and Rehabilitation, 25*(7), 331-342.

Bendix, A. F., Bendix, T., & Haestrup, C. (1998). Can it be predicted which patients with chronic low back pain should be offered tertiary rehabilitation in a functional restoration program? A search for demographic, socioeconomic, and physical predictors. *Spine, 23*(16), 1775-1783.

Bergstrom, G., Bergstrom, C., Hagberg, J., Bodin, L., & Jensen, I. (2010). A 7-year follow-up of multidisciplinary rehabilitation among chronic neck and back pain patients. Is sick leave outcome dependent on psychologically derived patient groups? *European Journal of Pain, 14*(4), 426-433. doi:http://dx.doi.org/10.1016/j.ejpain.2009.06.008

Bergstrom, G., Jensen, I. B., Bodin, L., Linton, S. J., & Nygren, A. L. (2001). The impact of psychologically different patient groups on outcome after a vocational rehabilitation program for long-term spinal pain patients. *Pain, 93*(3), 229-237.

Buchner, M., Neubauer, E., Zahlten-Hinguranage, A., & Schiltenwolf, M. (2007). Age as a predicting factor in the therapy outcome of multidisciplinary treatment of patients with chronic low back pain--a prospective longitudinal clinical study in 405 patients. *Clinical rheumatology, 26*(3), 385-392. doi:10.1007/s10067-006-0368-1

Burton, K., Polatin, P. B., & Gatchel, R. J. (1997). Psychosocial factors and the rehabilitation of patients with chronic work-related upper extremity disorders. *J Occup Rehabil, 7*(3), 139-153. doi:10.1007/bf02767360

Campello, M. A., Weiser, S. R., Nordin, M., & Hiebert, R. (2006). Work retention and nonspecific low back pain. *Spine, 31*(16), 1850-1857. doi:10.1097/01.brs.0000227288.00378.d5

Chapman, S. L., & Pemberton, J. S. (1994). Prediction of treatment outcome from clinically derived MMPI clusters in rehabilitation for chronic low back pain. *10*(4), 267-276.

Ciechanowski, P., Sullivan, M., Jensen, M., Romano, J., & Summers, H. (2003). The relationship of attachment style to depression, catastrophizing and health care utilization in patients with chronic pain. *Pain, 104*(3), 627-637. doi:10.1016/s0304-3959(03)00120-9

Corey, D. T., Koepfler, L. E., Etlin, D., & Day, H. (1996). A limited functional restoration program for injured workers: A randomized trial. *Journal of Occupational Rehabilitation, 6*(4), 239-249. doi:http://dx.doi.org/10.1007/BF02110886

Dobkin, P. L., Liu, A., Abrahamowicz, M., IonescuIttu, R., Bernatsky, S., Goldberger, A., & Baron, M. (2010). Predictors of disability and pain six months after the end of treatment for fibromyalgia. *The Clinical journal of pain, 26*(1), 23-29. doi:http://dx.doi.org/10.1097/AJP.0b013e3181b40ee6

Elfving, B., Asell, M., Luning Bergsten, C., & Alexanderson, K. (2010). Exploring activity limitations and sick leave among patients with spinal pain participating in multidisciplinary rehabilitation. *Disability & Rehabilitation, 32*(4), 292-299. doi:http://dx.doi.org/10.3109/09638280903095940

Elfving, B., Åsell, M., Ropponen, A., & Alexanderson, K. (2009). What factors predict full or partial return to work among sickness absentees with spinal pain participating in rehabilitation? *Disability & Rehabilitation, 31*(16), 1318-1327. doi:10.1080/09638280802572965

Fischer, C. A., Neubauer, E., Adams, H. S., Schiltenwolf, M., & Wang, H. (2014). Effects of multidisciplinary pain treatment can be predicted without elaborate questionnaires. *International orthopaedics, 38*(3), 617-626. doi:http://dx.doi.org/10.1007/s00264-013-2156-2

Gatchel, R. J., Mayer, T., Dersh, J., Robinson, R., & Polatin, P. (1999). The association of the SF-36 Health Status Survey with 1-year socioeconomic outcomes in a chronically disabled spinal disorder population. *Spine, 24*(20), 2162-2170.

Gatchel, R. J., Mayer, T. G., Kidner, C. L., & McGeary, D. D. (2005). Are gender, marital status or parenthood risk factors for outcome of treatment for chronic disabling spinal disorders? *Journal of Occupational Rehabilitation, 15*(2), 191-201.

Gatchel, R. J., Polatin, P. B., Mayer, T. G., & Garcy, P. D. (1994). Psychopathology and the rehabilitation of patients with chronic low back pain disability. *Archives of Physical Medicine & Rehabilitation, 75*(6), 666-670.

Glattacker, M., Heyduck, K., & Meffert, C. (2013). Illness beliefs and treatment beliefs as predictors of short-term and medium-term outcome in chronic back pain. *Journal of Rehabilitation Medicine, 45*(3), 268-276. doi:http://dx.doi.org/10.2340/16501977-1104

Grahn, B. E. M., Borgquist, L. A., & Ekdahl, C. S. (2004). Rehabilitation benefits highly motivated patients: A six-year prospective cost-effectiveness study. *International Journal of Technology Assessment in Health Care, 20*(2), 214-221.

Hampel, P., Graef, T., Krohn-Grimberghe, B., & Tlach, L. (2009). Effects of gender and cognitive-behavioral management of depressive symptoms on rehabilitation outcome among inpatient orthopedic patients with chronic low back pain: a 1 year longitudinal study. *18*(12), 1867-1880. doi:http://dx.doi.org/10.1007/s00586-009-1080-z

Hazard, R. G., Bendix, A., & Fenwick, J. W. (1991). Disability exaggeration as a predictor of functional restoration outcomes for patients with chronic low-back pain. *Spine (Phila Pa 1976), 16*(9), 1062-1067.

Heikkila, H., Heikkila, E., & Eisemann, M. (1998). Predictive factors for the outcome of a multidisciplinary pain rehabilitation programme on sick-leave and life satisfaction in patients with whiplash trauma and other myofascial pain: a follow-up study. *Clinical Rehabilitation, 12*(6), 487-496.

Hildebrandt, J., Pfingsten, M., Saur, P., & Jansen, J. (1997). Prediction of success from a multidisciplinary treatment program for chronic low back pain. *Spine, 22*(9), 990-1001.

Koopman, F. S., Edelaar, M., Slikker, R., Reynders, K., van der Woude, L. H., & Hoozemans, M. J. (2004). Effectiveness of a multidisciplinary occupational training program for chronic low back pain: a prospective cohort study. *American journal of physical medicine & rehabilitation / Association of Academic Physiatrists, 83*(2), 94-103. doi:10.1097/01.PHM.0000107482.35803.11

Lillefjell, M., Krokstad, S., & Espnes, G. A. (2007). Prediction of function in daily life following multidisciplinary rehabilitation for individuals with chronic musculoskeletal pain; a prospective study. *BMC musculoskeletal disorders, 8*, 65. doi:10.1186/1471-2474-8-65

Ljungkvist, I. (2000). Short- and long-term effects of a 12-week intensive functional restoration programme in individuals work-disabled by chronic spinal pain. *Scandinavian Journal of Rehabilitation Medicine - Supplementum, 40*, 1-14.

Martin, J., Torre, F., Padierna, A., Aguirre, U., Gonzalez, N., Matellanes, B., & Quintana, J. M. (2014). Interdisciplinary Treatment of Patients with Fibromyalgia: Improvement of Their Health-Related Quality of Life. *Pain Pract., 14*(8), 721-731. doi:10.1111/papr.12134

Mentari, A., Atwood, G. T., Reger, S. I., & Levin, T. Z. (1999). Effect of participation in a pain and stress management program on return-to-work outcome. *American Journal of Pain Management, 9*(1), 28-34.

Michaelson, P., Sjolander, P., & Johansson, H. (2004). Factors predicting pain reduction in chronic back and neck pain after multimodal treatment. *Clinical Journal of Pain, 20*(6), 447-454. doi:http://dx.doi.org/10.1097/00002508-200411000-00010

Moradi, B., Hagmann, S., Zahlten-Hinguranage, A., Caldeira, F., Putz, C., Rosshirt, N., . . . Neubauer, E. (2012). Efficacy of Multidisciplinary Treatment for Patients With Chronic Low Back Pain A Prospective Clinical Study in 395 Patients. *Jcr-Journal of Clinical Rheumatology, 18*(2), 76-82. doi:10.1097/RHU.0b013e318247b96a

Nyberg, V. E., Novo, M., & Sjolund, B. H. (2011). Do Multidimensional Pain Inventory scale score changes indicate risk of receiving sick leave benefits 1 year after a pain rehabilitation programme? *Disability & Rehabilitation, 33*(17-18), 1548-1556. doi:http://dx.doi.org/10.3109/09638288.2010.533815

Orenius, T., Koskela, T., Koho, P., Pohjolainen, T., Kautiainen, H., Haanpaa, M., & Hurri, H. (2013). Anxiety and depression are independent predictors of quality of life of patients with chronic musculoskeletal pain. *Journal of Health Psychology, 18*(2), 167-175. doi:http://dx.doi.org/10.1177/1359105311434605

Persson, E., Lexell, J., Eklund, M., & Rivano-Fischer, M. (2012). Positive effects of a musculoskeletal pain rehabilitation program regardless of pain duration or diagnosis. *Pm & R, 4*(5), 355-366. doi:http://dx.doi.org/10.1016/j.pmrj.2011.11.007

Persson, E., Lexell, J., Rivano-Fischer, M., & Eklund, M. (2014). Occupational performance and factors associated with outcomes in patients participating in a musculoskeletal pain rehabilitation programme. *Journal of Rehabilitation Medicine, 46*(6), 546-552. doi:http://dx.doi.org/10.2340/16501977-1810

Polatin, P. B., Cox, B., Gatchel, R. J., & Mayer, T. G. (1997). A prospective study of Waddell signs in patients with chronic low back pain. When they may not be predictive. *Spine, 22*(14), 1618-1621.

Salgueiro, M., Basogain, X., Collado, A., Torres, X., Bilbao, J., Donate, F., . . . Azkue, J. J. (2013). An Artificial Neural Network Approach for Predicting Functional Outcome in Fibromyalgia Syndrome after Multidisciplinary Pain Program. *Pain Medicine (United States), 14*(10), 1450-1460. doi:http://dx.doi.org/10.1111/pme.12185

Tollison, C. D. (1993). Compensation status as a predictor of outcome in nonsurgically treated low back injury. *Southern Medical Journal, 86*(11), 1206-1209.

Trief, P. M., & Yuan, H. A. (1983). The use of the MMPI in a chronic back pain rehabilitation program. *Journal of Clinical Psychology, 39*(1), 46-53.

van Hooff, M. L., Spruit, M., O'Dowd, J. K., van Lankveld, W., Fairbank, J. C., & van Limbeek, J. (2014). Predictive factors for successful clinical outcome 1 year after an intensive combined physical and psychological programme for chronic low back pain. *European Spine Journal, 23*(1), 102-112. doi:http://dx.doi.org/10.1007/s00586-013-2844-z

Watson, P. J., Booker, C. K., Moores, L., & Main, C. J. (2004). Returning the chronically unemployed with low back pain to employment. *European Journal of Pain, 8*(4), 359-369. doi:10.1016/j.ejpian.2003.11.003

Vendrig, A. A. (1999). Prognostic factors and treatment-related changes associated with return to work in the multimodal treatment of chronic back pain. *Journal of Behavioral Medicine, 22*(3), 217-232.

Verkerk, K., Luijsterburg, P. A. J., Heymans, M. W., Ronchetti, I., Pool-Goudzwaard, A. L., Miedema, H. S., & Koes, B. W. (2015). Prognosis and course of pain in patients with chronic non-specific low back pain: A 1-year follow-up cohort study. *European Journal of Pain (United Kingdom), 19*(8), 1101-1110. doi:http://dx.doi.org/10.1002/ejp.633

Wilson, H. D., Mayer, T. G., & Gatchel, R. J. (2011). The lack of association between changes in functional outcomes and work retention in a chronic disabling occupational spinal disorder population: Implications for the minimum clinical important difference. *Spine, 36*(6), E474-E480. doi:http://dx.doi.org/10.1097/BRS.0b013e3181d41632
